# *Macrocheles kekensis* sp. n., a new macrochelid mite associated with a centoniin beetle from Hungary (Acari, Mesostigmata)

**DOI:** 10.3897/zookeys.768.24460

**Published:** 2018-06-19

**Authors:** Jenő Kontschán

**Affiliations:** 1 Plant Protection Institute, Centre for Agricultural Research, Hungarian Academy of Sciences, H-1525 Budapest, P.O. Box 102, Hungary

**Keywords:** Acari, Coleoptera, phoresy

## Abstract

A new species, *Macrocheles kekensis* **sp. n.**, is described based from three specimens associated with a cetoniin beetle (*Hoplia hungarica* Burmeister, 1844). The new species differs from the other known European macrochelid species in having 29 pairs of dorsal setae, j1 and z1 short and robust, other dorsal setae long and pilose, and the absence of apodemes between the genital and ventrianal shields. This is the 34^th^ Hungarian macrochelid species.

## Introduction

Members of the mite family Macrochelidae (Parasitiformes: Mesostigmata) are large, fast-moving predators inhabiting soil substrates, litter and decomposing organic matter. The macrochelids feed on nematodes, eggs and larvae of insects or weakly sclerotized mites, and very often live in association with certain insect groups (e.g. flies and beetles) (Mašán 2003). Macrochelidae is relatively well known in some Central European countries like Germany (Karg 1993), Austria (Johnston 1970) and Slovakia (Mašán 2003). However, information about their occurrence in Hungary is insufficient, although numerous records were presented over the past 20 years (e.g. Ács and Kontschán 2014, Kontschán 2005, 2006, 2015, Kontschán et al. 2014, 2015a, 2015b, 2016, Salmane and Kontschán 2005, 2006). Only a few beetle-associated macrochelids have been mentioned (Kontschán 2006) from Hungary, while macrochelid mites associated with centoniin beetles are rarely collected. The association of macrochelid mites with flower beetles seems to be a rare phenomenon. Mašán (2003) in his monograph about the macrochelid mites of Slovakia mentioned only four species associated with a centoniin species (*Potosia cuprea* Fabricius, 1775).

The subfamily Cetoninae is a very species-rich group in Hungary (Enyedi 2006), majority of the species can be observed on the flowers of the plants in summertime. So far only one species [*Macrocheles glaber* (J. Müller, 1860)] has been reported from *Cetonia aurata* from Hungary (Ács and Kontschán 2014). Recently, some macrochelids were collected on a *Hoplia hungarica* Burmeister, 1844 beetle (Scarabaeidae: Cetoniinae), which are described as a new species here.

## Materials and methods

The three mite specimens were collected as phoretic individuals on a *Hoplia hungarica* Burmeister, 1844 beetle in the eastern part of Hungary. The host beetle was attracted to a lamp of the house and was found on the ground close to the house wall. The specimens assigned here to the new species (n = 3, females) were collected from the body of the host beetle using a brush observed under a BTC binocular microscope. Later they were cleared in lactic acid and were placed on a slide with deep cavity for examination. Drawings were made with the aid of a drawing tube on a Leica 1000 microscope. All specimens are stored in 75% ethanol and the holotype and one paratype are deposited in the Natural History Museum, Budapest, plus one paratype in the Natural History Museum in Geneva. Measurements are presented in minimum and maximum size. Measurements and the scales in the figures are given in micrometers (μm). The new species was also tested using the keys provided by Bregetova (1977), Karg (1993) and Mašán 2003.

## Taxonomy

### Macrochelidae Vitzthum, 1930

#### *Macrocheles* Latreille, 1829

##### Macrocheles kekensis sp. n.

Taxon classificationAnimaliaMesostigmataMacrochelidae

http://zoobank.org/7E437D0D-B6D5-4530-9388-1C2AE23D955B

Figures 1–2 , 3–7 , 8–15

###### Diagnosis.

All dorsal setae pilose, except setae j1 and z1 which short and spine-like. Anterior and lateral parts of dorsal shield dotted, majority of dorsal surface with reticulate sculptural pattern.

###### Material examined.

Holotype. Female. Collected from *Hoplia hungarica* Burmeister, 1844 Hungary, Kék village, 48°06'38"N, 21°52'51"E, 10 m a.s.l., 05 May 2017. Kontschán, J. coll. *Paratypes.* Two females, locality, date and host same as for holotype. The holotype and one paratype were deposited in the Soil Zoology Collections of the Hungarian Natural History Museum, Budapest, the other paratype in the Arachnida collection of the Natural History Museum, Geneva, Switzerland.

###### Description.

**Figures 1–2.**
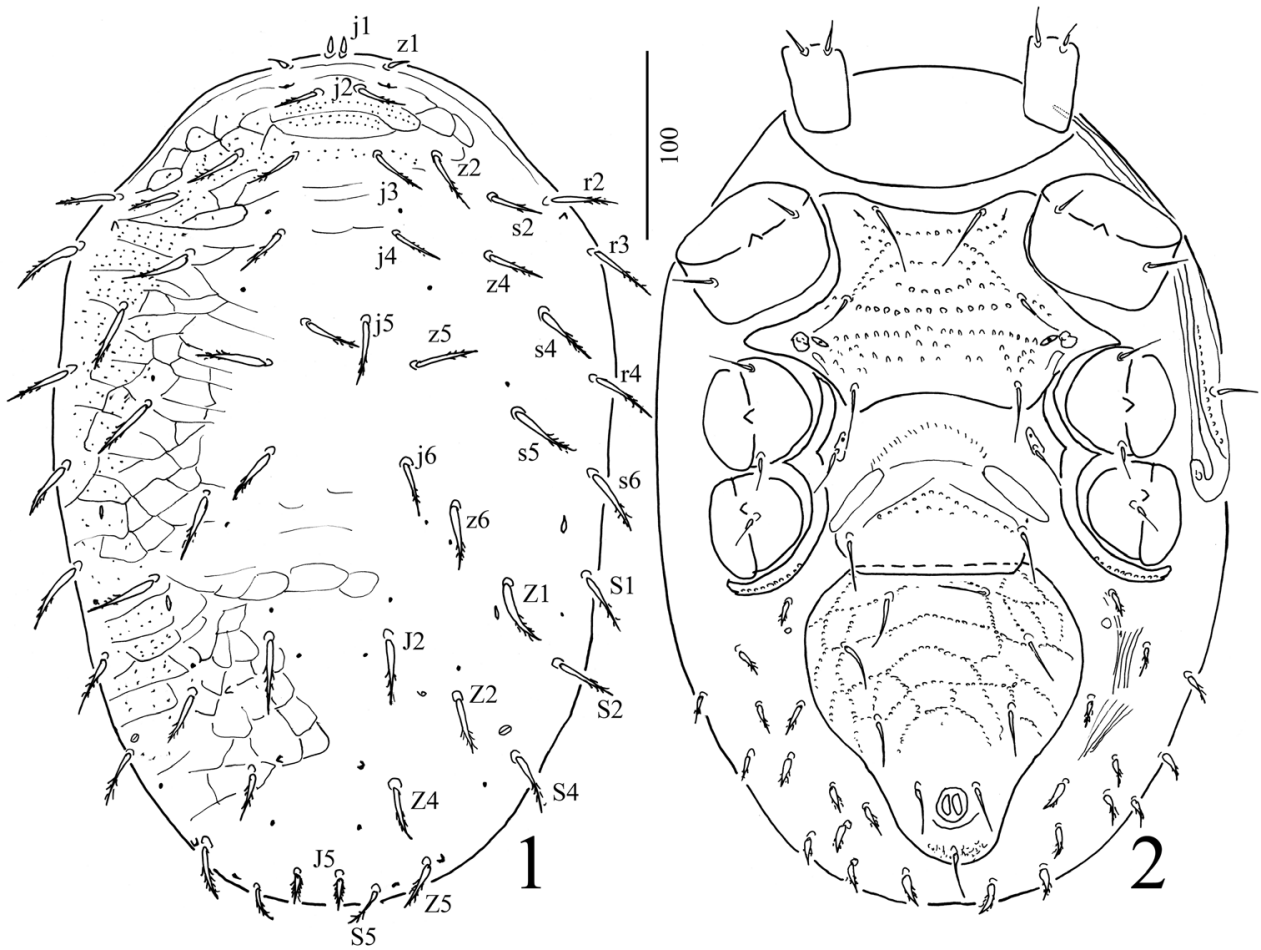
*Macrocheles kekensis* sp. n., female. **1** Dorsal view of body **2** Ventral view of body (without legs and gnathosoma, only the coxae illustrated).

Female. *Dorsum* (Figure 1). Shape of dorsal shield oblong, with length 440–460 and width 280–310 at level of the coxae II (n=3), micropunctation on anterior and lateral surface with reticulate sculptural pattern. Majority of setae on dorsal shield long and pilose in distal half, except setae j1 and z1 which are short, smooth and spine-like, and J5 with pilose margins for entire length. Length of dorsal setae: j1 and z1= 8–9, j2 = 22–24, j2, s2, Z2, Z5 and S5 = 24–26, j3, j4, j6, z2, z4, z5, z6, s4, s6, J2, Z1, Z4, S1, S2, S4 = 30–32, j5, s5, s6, r3, r3, r4 = 34–35, J5 = 16. Dorsal shield with four pairs of lyrifissures (close to z1, s6, S5 and between Z2 and S4), ten pairs of gland pores (close to j3, j4, s5, j6, z6, Z1, Z2, S4, Z4 and two close to J2), and four pairs of microspicules (close to r2, Z2, Z4 and Z5). Peritreme reaches the bases of setae z1.

*Venter* (Figure 2). Sternal shield 87–95 long and 100–110 wide at level of coxae II, bearing 3 pairs of needle-like setae and two pairs of lyrifissures. Posterior margin of sternal shield concave. Surface with linearly arranged punctures. Measurements of setae: St1 = 34–36, St2 = 30–32 and St3 = 26–28. Cuticle with a thin punctuation. Metasternal seta on metasternal platelet with length 20–23. Posterior margin of genital shield straight. Genital shield with length 75–78 and width 89–93. The length of seta on genital shield 23–26. Apodemes between ventrianal and genital shields absent. Ventrianal shield pentagonal with length 158–162 and width at level of Jv2 145–148, surface reticulated with linearly arranged punctures. Three pairs of ventrianal setae 22–25 long, needle-like, adanal setae needle-like and 22 long. Post-anal seta similar in shape and length to adanal setae. Opisthogaster bearing more than twelve pairs of slightly pilose setae (12–19 long). Sperm access system not visible.

**Figures 3–7.**
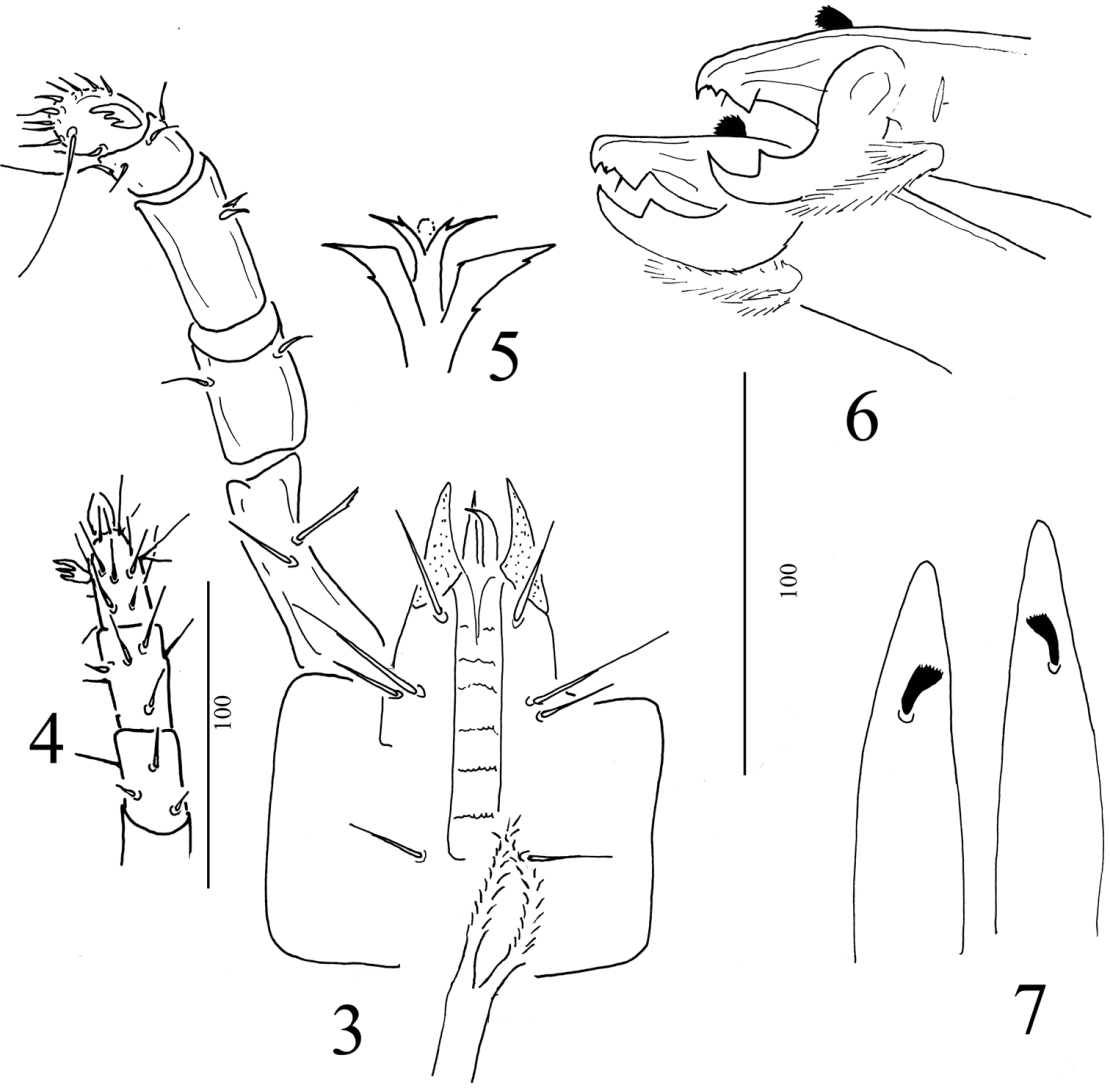
*Macrocheles kekensis* sp. n., female. **3** Ventral view of gnathosoma and palp **4** Palp in dorsal view **5** Tectum **6** Dorsal view of chelicerae **7** Lateral view of chelicerae.

*Gnathosoma* (Figures 3–7). Gnathosoma well developed. Deutosternal groove (Figure 3) with six rows of denticles, 3 pairs of hypostomal setae and one pair of palpcoxal setae present, all setae needle-like. Internal posterior hypostomal seta (h2) longer than others. Measurements of hypostomal setae: h1 = 27–30, h2 = 38–42, h3 = 18–25 and capitulate seta = 20–27. Tectum (Figure 5) with a pair of lateral processes and a bifurcated medial stem, margins of lateral processes and medial stem weakly serrate. Cheliceral fixed digit with apically serrate dorsal seta, four teeth (two smaller and two larger), pilus dentilis and terminal hook (Figures 6–7). On moveable digit large tooth and terminal hook present. Arthrodial brush with a short and a long branch and densely pilose. Length of fixed digit 52–55, moveable digit 41–44. Palp 164–170 long, palp trochanter with one smooth and one apically serrate ventral setae, other setae on palp smooth, palp apotele three-tined (Figures 3–4).

*Legs* (Figures 8–15). Tarsi II–IV with well-developed ambulacra and claws, claws and ambulacrum is missing from the tip of leg I. All setae simple on legs. Leg chaetotaxy typical for the genus. Length of legs: I 325–370, II 250–320, III 240–260, IV 380–420. Coxal glands not visible.

###### Etymology.

The name of the new species refers to the village (Kék, East-Hungary) where the species was collected.

**Figures 8–15.**
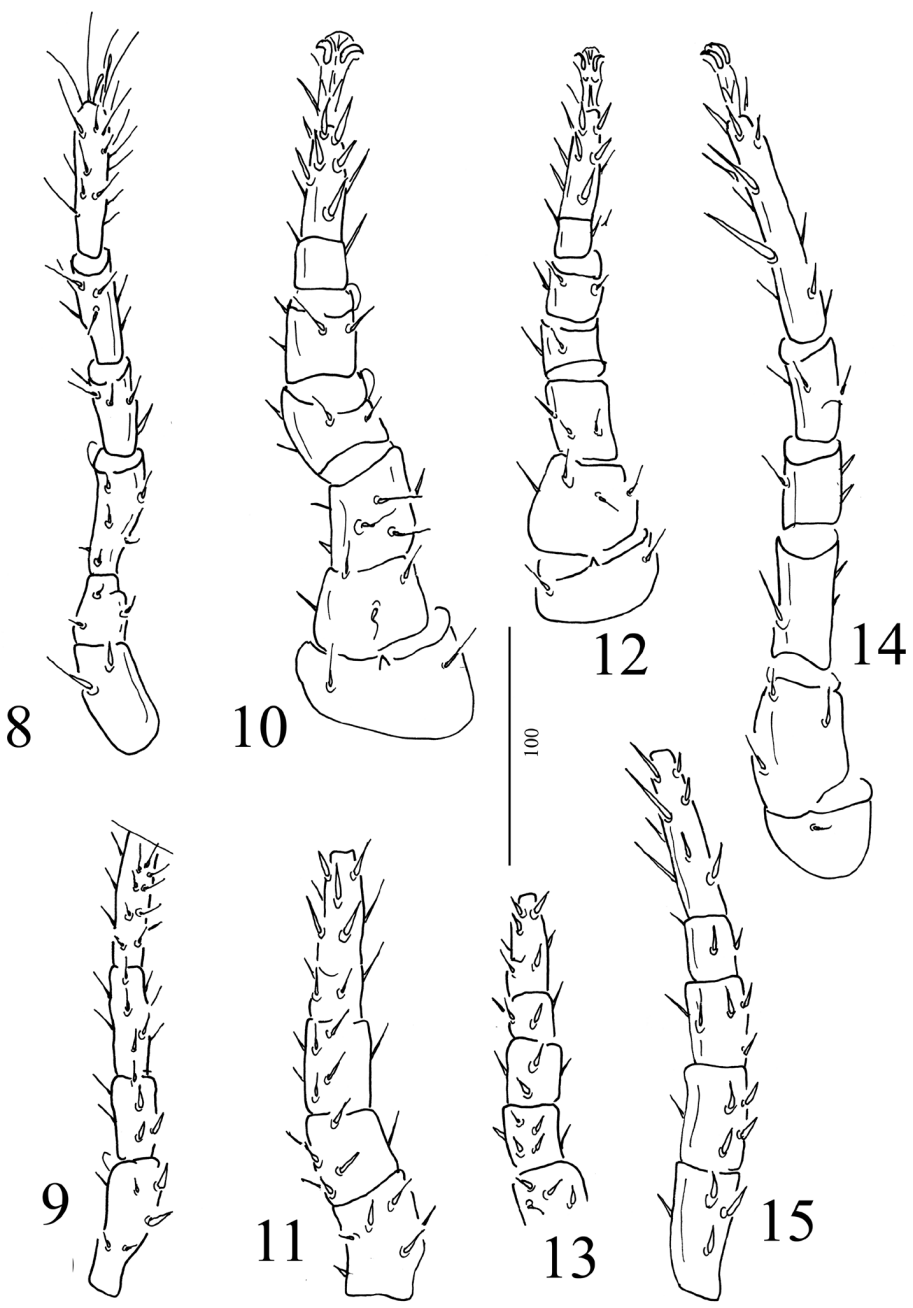
*Macrocheles kekensis* sp. n., female **8** Leg I in ventral view **9** Leg I in dorsal view **10** Leg II in ventral view **11** Leg II in dorsal view **12** Leg III in ventral view **13** Leg III in dorsal view **14** Leg IV in ventral view **15** Leg IV in dorsal view.

## Remarks

The short, robust, and small setae j1 and z1 are present in numerous macrochelid mites distributed in Europe, but the majority or all dorsal setae are smooth in these known species, contrary with new one, where only the latter mentioned two pairs of setae are smooth and the others are marginally pilose. *Macrocheles subbadius* (Berlese, 1904) and *Macrocheles insignitus* Berlese, 1918 have similar ornamentations on sternal and ventral shields and have short and robust j1 setae, but these two species have smooth setae on dorsal shield, which are pilose in the new one.

## Discussion

The systematic position of the new species is questionable. The serrate dorsal seta on chelicerae are a distinctive character of the genus *Nothrholaspis*, but members of the genus *Nothrholaspis* has three pairs of small apodemes between genital and ventrianal shields and tectums are forked to lateral and central branches (Emberson 2010, Babaeian et al. 2014, Özbek and Bal 2013). The dorsal seta of the chelicera of the new species is apically serrate, which matches the diagnosis of the genus *Nothrholaspis*. However, the apodemes are missing in the new species and the shape of tectum is also different. Due to the shape of tectum and the absence of apodemes between the genital and ventrianal shield, I cannot place the new species into the genus *Nothrholaspis*, therefore it is temporarily placed in the genus *Macrocheles* sensu lato.

The host species (*Hoplia hungarica*) is a rare beetle found in Hungary. Usually only one or two localities are mentioned in faunistic studies (Ádám 1997, Enyedi 2006, Rozner 2001). Therefore the finding of the macrochelid mite on this infrequently collected beetle was absolutely unexpected.

## Supplementary Material

XML Treatment for Macrocheles kekensis
